# Time to initiate postpartum modern contraceptive use and predictors among women of reproductive age group in Dilla Town, Southern Ethiopia: a retrospective cohort study

**DOI:** 10.1186/s40834-022-00189-6

**Published:** 2022-10-02

**Authors:** MaeregayehuTibo, Atnafu Adem, Azmach Dache

**Affiliations:** 1 Dilla University College of Health Sciences, Addis Ababa, Ethiopia; 2 Department of Reproductive Health, Dilla University, College of Health Sciences, Dilla, Ethiopia; 3 Social and Population Health Department, Yirgalem Hospital Medical College, Addis Ababa, Ethiopia; 4 Social and Population Health Department, Yirgalem Hospital Medical College, Yirgalem, Ethiopia

**Keywords:** Time to initiate, Modern contraception use, postpartum period, Ethiopia

## Abstract

**Background:**

Globally 1 in 7 women aren’t using family planning as a result, larger numbers of women get pregnant within 7–9 months of childbirth. The aim of this study was to estimate the time to initiate postpartum modern contraceptive use and predictors among women of reproductive age group within the first 12 months of delivery in Della Town, Southern, Ethiopia.

**Methods:**

A retrospective cohort study from March 25, 2019, to March 25, 2020, was conducted in Dilla town. A Systematic sampling technique was used to select 594 study participants. A Cox proportional hazards model was used to determine factors associated with time to initiate postpartum modern contraceptive use at 95% CI with a *P*-value of < 0.05.

**Results:**

A total of 576 postpartum women were participated making a response rate of 96.9%. The median time to initiate postpartum modern contraceptive use was 7 months (IQR: 6, 8). Education [AHR = 3.01 (95% CI = 1.32, 6.83)], knowledge on family planning [AHR = 1.56(95% CI = 1.20, 2.02)], and family planning counseling during postnatal care [AHR = 2.22 (95% CI = 1.46, 3.38)] were predictors positively associated with time to initiate postpartum modern contraceptive.

**Conclusions:**

The time to initiate postpartum modern contraceptive use was delayed longer than compared to the World Health Organization recommendation. Education level of women, knowledge of family planning, and family planning counseling during postnatal care were some predictors positively associated with time to initiate postpartum modern contraceptive use.

## Introduction

Postpartum family planning (PPFP) is defined as the prevention of unintended pregnancy and closely spaced pregnancies through the first 12 months following childbirth [1]. During the postpartum period family planning (FP) can prevent about 30% of maternal mortality and 10% of child mortality if couples space their pregnancies more than 2 years apart [2]. World health organization (WHO) recommended time for the initiation of contraceptives in the postpartum period is 6 weeks after delivery [3].

Globally, more than 90% of women during the first year of the postpartum period want to either delay or avoid future pregnancies. However, 70% are not using 29 PPFP. In most cases, sexual activity in the postpartum is resumed before the menstruation following delivery without the use of any contraceptive method [1]. Worldwide, there are 265 million unwanted pregnancies, 110 million unnecessary abortions, 590,000 avoidable maternal deaths, and 8 million preventable infant deaths [4]. According to the analysis done in 172 countries 33 without family planning use the number of maternal deaths would have been 1.8 times higher (equivalent to 614,000 deaths) than with family planning use which means that contraceptive use averted 44.3% of maternal deaths [5].

The WHO reports that over 60% of maternal deaths in developing countries occur during this postpartum period [6]. A woman in a developing country is 97 times more likely to die as a result of pregnancy than a woman in a developed country. The developing regions share approximately 99% of the estimated global maternal deaths in 2015 and out of this, 66% were from Sub-Saharan Africa (SSA) [6]. An estimated 30 million unplanned births and 40 million abortions, half of them illegal and unsafe, occurred annually in low and middle-income countries [7].

In SSA, 40% of married women do not want a child in the next 2 years but are not using contraception. As a consequence, nearly 25% of pregnancies in the region are unintended [8]. A demographic and health survey (DHS) data from 57 countries indicated that, right after delivery 62%, after 6 months of amenorrhea 43% and at the end of amenorrhea 32% of women in the first year after birth have an unmet need for contraception [9].

Despite all these efforts done the utilization of modern contraceptives is low special during the postpartum period. Study shows Ethiopia that about 86% of the women in Ethiopia have an unmet need during the first year of the postpartum period [10, 11]. Therefore, the purpose of this study is to estimate the time to initiate postpartum modern contraceptive use and predictors among women of reproductive age group within the first 12 months of delivery in Dilla town, southern, Ethiopia.

## Methods

### Study area, design, and period

The study was conducted at Dilla Town, which is the administrative center of the Gedeo zone in the Southern region of Ethiopia. It is 365 km away from Addis Ababa capital city of Ethiopia and 95 km from Hawassa the administrative center of the southern region of Ethiopia and the main road from Addis Ababa to Nairobi, Kenya crosses the town. The town is surrounded in the north by Sidama, in the south by Wonago, in the east by Bule and Oromia and in the west by Oromia regional state. Administratively the town is divided into 9 Kebeles (small local administrative units of Ethiopia). According to the 2007 census conducted by the Central Statistical Agency of Ethiopia (CSA); Dilla town has a total population of 102,624 in 2016 as projected from the 2007 Census among these 50,286 (48.9%) are males & 52,338 (51.1%) are females and the total number of women in the reproductive age group is 23,911 and the total number delivery is 3274. A community-based retrospective cohort study was conducted among women of the reproductive age group who gave birth 12 months prior to the study in Dilla town, southern, Ethiopia from March 25, 2019, to March 25, 2020.

### Study population and sample size determination

All women of the reproductive age group who gave birth 12 months prior to the study in Dilla town were source population; women of the reproductive age group who gave birth 12 months prior to the study in randomly selected kebeles in Dilla town were study population; women of the reproductive age group who gave 12 months prior to the study and residing for at least 12 months in the Dilla town were included in the study; women of the reproductive age group who gave 12 months prior to the study but critically ill and unable to respond during the data collection period and not residing for at least 12 months in Dilla town were excluded from the study. The sample size was calculated by using the double population proportion formula by Epi Info 78 version 7 software. We took the maximum sample among the most significant predictors of time to initiate postpartum modern contraceptive use in most work of literature [12–14]. Unfortunately, a study done in Kenya factor place of delivery was selected as an independent variable since it gave maximum sample size as compared to other exposure variables. The calculation was based on the assumption that 95% confidence level, 80% power, the ratio of non-exposed to exposed 1, outcome variable among exposed 49% and non-exposed 24.7%. The highest sample size was 360 then after considering 10% non-response rate and 1.5 design effects finally sample size was 594 used for this study.

### Sampling procedure

A Systematic sampling technique was used to select study participants. Nine [9] kebeles were found in Dilla Town, from these randomly 5 Kebeles were selected. In 5 selected kebeles census was done to identify households where the women found who gave birth 12 months before the study period in each selected kebeles reside and identification number was given for households with eligible women. A sampling frame was developed for each selected kebele separately based on the result of censes. Then calculated sample size was proportionally allocated to each selected Kebele based on its number of eligible women. Then study participants were selected by systematic sampling techniques. The sampling interval was obtained by dividing the total number of postpartum women in each of Kebele by the proportionally allocated sample of each Kebele. The first postpartum women was selected by lottery method. Every 2 postpartum woman was included until the required sample size for each Kebele was achieved. If the selected postpartum woman was absent at the time of data collection, the data collectors revisited for two consecutive times, and if the interviewers failed to find the study participant after two visits, the next postpartum woman was included in the study.

### Data collection procedure and quality control

Data were collected by using interviewer-administered structured and pretested questionnaire. The questionnaire was adapted from different related literature in such a way it includes all relevant variables to meet the objective of the study [15–18]. The questionnaire consisted of six pages and five of the sections were tries to cover: socio-demographic characteristics, reproductive health-related characteristics, Contact with a health care professionals, knowledge on FP, and time to initiate postpartum modern contraceptive use. The questionnaire was first prepared in English and translated into Amharic and Gedeofa (local language) by language experts. Data was collected by 4 data collectors who have diploma midwifery and 2 supervisors who have Bachelor of Science nurse (BSc) were involved. Interview conducted in each study participants’ home.

To maintain data quality properly designing, translating and pre-testing the questionnaire was done. Data was collected by 4 data collectors who have diploma midwives and could speak the local language (Gedeofa) and 2 supervisors who have BSc in nursing working in the study area. Data was collected after giving Two-day proper training on the interviewing techniques, the importance of privacy, discipline, and confidentiality of the study participant. The pretest was done 1 week prior to the actual data collection time on 5% of the total sample size [19] at Chuchokebele in Dilla Zuriya woreda which is outside of the study area. It was done to see for the exactness of the responses for the questions asked, language clarity, and appropriateness of the tools before the actual data collection was conducted. Based on the results of the pretest, the time required for interviewing each participant was estimated and the skip pattern of some of the questions was corrected. Closer supervision was made to check the data collectors whether they are in their work and interview the right women who were included in the study. Confusions and questions were solved by the supervisors and principal investigator during data collection.

### Study variables

Time to initiate postpartum modern contraceptive use was a dependent variable, and the independent variables are socio-demographic and economic characteristics: age of women, marital status, educational status of women, occupation status of women, and wealth status; Contact with a health care professional: Antenatal Care visit (ANC), Number of ANC visit, Postnatal care visit (PNC), Place of delivery, Distance to the health facility, FP counseling during ANC, FP counseling during PNC, Child immunization and FP counseling during child immunization; Reproductive health character: Parity, birth interval, fertility desire, resume sexual intercourse, resume menses, discuss with the partner on FP, who decide on PPFP use and previous use of modern contraceptive, Knowledge of Postpartum family planning.

### Operational definition

Time to initiate postpartum modern contraceptive use: Time to initiate postpartum modern contraceptive use was calculated at the time between the date of giving birth to the date of initiating postpartum modern contraceptive use (in the month) [20] . Event: initiation of postpartum modern contraceptive use. Censored: study participant who lost to follow-up and not initiate postpartum modern contraceptive use up to the end of the study period. Good knowledge: is defined as those study participants who score yes 4 knowledge on PPFP related question was good knowledge [16, 21] . Poor knowledge: is defined as a study participant score yes less than < 4 knowledge on PPFP related question was poor knowledge [16, 21]. Pills users: postpartum women in the reproductive age group who are continuously using progesterone-only pill (POP) for 1 month period and longer.

### Data processing and analysis

The tool’s completeness was verified prior to data entry, and the data were coded, entered, and stored in Epi Data version 3.1 before being analyzed in Statistical Package for Social Science software version 20 (SPSS). Descriptive statistics were done for categorical variables. Continuous variables were expressed as a medians and inter-quartile ranges. Wealth status was measured by the wealth index generated from the household’s cumulative living standard based on ownership of specified assets using factor analysis and was later categorized into quintiles (Lowest, Second, Middle, Fourth, and Highest). The life table was constructed to estimate the overall probability of the initiation of postpartum modern contraceptives over time. A Kaplan Meier survival curve was used to estimate the time to initiation of postpartum modern contraceptive use among different groups of variables. A Log rank test was used to compare survival curves between different categories of explanatory variables. A bivariate and multivariable Cox proportional hazards model was used to assess the independent variable associated with time to initiate postpartum modern contraceptive use. Variables with a *p*-value of < 0.25 in the bivariate analysis were fitted in to multivariate analysis. The final model was checked for satisfying the assumption of proportionality with the time-dependent Cox model and graphically by the log-log hazard plot and the proportional hazard assumption was not violated. The model fitness was also checked using Cox and Snell’s residual analysis. An adjusted hazard ratio (AHR) with a 95% confidence interval (CI) was calculated to estimate the strength of the association between independent predictors and the time to initiate postpartum modern contraceptive use. Statistical significance was defined as a *p* value of 0.05.

## Results

### Socio-demographic characteristics of study participants

From 594 study participants planned for interview, about 576 respondents were interviewed making a response rate of 96.9%. The mean age of the respondents was 28.9 (SD 5.4). Regarding respondents marital status, religion, ethnicity, occupation, and education, 403 (70%) were married, 324 (56.2%) were protestant followers, 274 (47.6%) were Gedeo, 280 (48.6%) were housewives, and 289 (50.2%) attended primary education. Regarding wealth status, 137 (23.8%) were in the second wealth quintile (Table 1).

**Table 1:** Socio-demographic characteristics of study participants in Dilla Town, southern, Ethiopia, 2020

Variables	Frequency	Percent
**Age of the women**		
19–24	160	27.8%
25–34	278	48.2%
≥ 35	138	24.0%
**Marital status**		
Married	403	70%
Single	133	23.1%
(divorced & widowed)	40	6.9%
**Religion**		
Protestant	324	56.3%
Orthodox	159	27.7%
Muslim	49	8.5%
Catholic	33	5.6%
Others	11	1.9%
**Ethnicity**		
Gedeo	274	47.6%
Oromo	90	15.6%
Sidama	74	12.9%
Gurage	101	17.5%
Others	37	6.4%
**Education level of women**		
No formal education	35	6.1%
Read and write	40	6.9%
Primary completed	289	50.2%
Secondary and above	212	36.8%
**Occupation status of women**		
Housewife	280	48.6%
Government employee	134	23.3%
Private employee	77	13.4%
Merchant	75	13.0%
Others (Daily laborer & student)	10	1.7%
**Wealth status**		
Lowest	107	18.6%
Second	137	23.8%
Middle	113	19.6%
Fourth	96	16.7%
Highest	123	21.3%

### Reproductive characteristics of study participants

The average number of children per woman was two. Among the study participants, 485 (84.2%) had recent child birth alive. Approximately 178 (30.9%) of study participants had a gap of more than 2 years between their previous and current births. Majority of the respondents 472 (81.9%) had a previous history of FP use, and the rest of respondents had no history of previous FP use. Reason for not using 24 (4.2%): fear of side effects. The majority of respondents (409, or 71.0%) want another child after 2 years. Of respondent 351 (60.9%) did not discuss with their partner family planning. The majority of study participants 439 (76.2%), had resumed menses and the median time was 9 weeks, and 398 (69.1%) of the study participants had already 184 resumed sexual intercourse at the median time of 11 weeks (Table 2).

**Table 2:** Reproductive related characteristics of study participants in Dilla Town, southern Ethiopia, 2020

Variables	Frequency	Percent
**Parity**		
1	150	26.0%
2–3	274	47.6%
4+	152	26.4%
**Birth interval**		
It is my first child	150	26.1%
< 12 months	37	6.4%
12–24 months	211	36.6%
> 24 months	178	30.9%
**Status of recent birth**		
Alive	485	84.2%
Died	91	15.8%
**Previous history of modern family planning use**		
No	104	18.1%
Yes	472	81.9%
**Reason for not use Previously modern family planning**		
fear of side effects	24	4.2%
husband disproval	14	2.4%
in breastfeeding	9	1.6%
menses not return	14	2.4%
far distance heath facility	11	1.9%
want deliver soon	13	2.3%
Others	19	3.3%
**Future desire**		
Want before 2 years	91	15.4%
Want after 2 years	409	71.0%
Want no more children	76	13.6%
**Discus with partner on family planning use**		
No	351	60.9%
Yes	225	39.1%
**Who decide on family planning use**		
Respondent (women)	214	37.2%
Husband	137	23.8%
Both	225	39.0%
**Menses resumption after recent birth**		
No	137	23.8%
Yes	439	76.2%
**Months menses resumed**		
Less than 3 months	431	98.2%
3–5 months	3	0.7%
Greater than 5 months	5	1.1%
**Sexual intercourse resumed after recent birth**		
No	178	30.9%
Yes	398	69.1%
**Months Sexual intercourse resumed**		
Less than 3 months	308	77.4%
3–5 months	82	20.6%
Greater than 5 months	8	2.0%

### Contact of study participants with a health care professional

Almost all 559(97.0%) study participants received ANC service, with 444(81.1%) receiving two or more ANC visits during pregnancy and361 (64.6%) receiving FP counseling during ANC service. About 470(81.6%) of mothers gave birth to their current child in governmental and private health institutions with health professionals. Almost all of the 556(96.5%) and 517 (89.8%) respondents were born via spontaneous vaginal delivery or as a single child, respectively. About 364(63.2%) of respondents had gotten PNC service; of these 251(69.0%) of respondents had family planning counseling during the postnatal period. The majority of respondents 518 (89.9%) went health facility for child immunization, butonly206, (39.8%) get family planning counseling during child immunization (Table 3).

**Table 3:** Contact of study participants with Health Care Professional in Dilla Town, southern, Ethiopia, 2020

Variables	Frequency	Percent
**ANC visit for recent pregnancy**		
No	17	3.0%
Yes	559	97.0%
**Number of ANC visit**		
1	115	20.3%
2–3	299	53.8%
4+	145	25.9%
**FP counseling during ANC service**		
No	198	35.4%
Yes	361	64.6%
**Place of delivery recent birth**		
Government health facility	362	62.8%
Private health facility	108	18.8%
Home	106	18.4%
**Distance to health facility**		
Less than half hours	108	18.8%
Half hour-1 hour	305	53.0%
Greater than 1 hours	163	28.2%
**Birth attendants for recent birth**		
Health professionals	463	80.4%
Non health professionals (TBA)	101	17.5%
Others (relatives)	12	2.1%
**Mode of delivery for recent birth**		
Spontaneous vaginal delivery	556	96.5%
Caesarian section	20	3.5%
**Type of birth for recent birth**		
Single	517	89.8%
Multiple (twins)	59	10.2%
**Postnatal care visit for recent birth**		
No	212	36.8%
Yes	364	63.2%
**FP counseling during postnatal care service for recent birth**		
No	113	31.0%
Yes	251	69.0%
**Gone to health facility for child immunization**		
No	58	10.1%
Yes	518	89.9%
**FP counseling during child immunization**		
No	312	60.2%
Yes	206	39.8%

### Knowledge of post partum FP of study participants

Among the respondents, only 242 (42%) had good knowledge. Whereas the majority 334 (58%) of the respondents had poor knowledge of post partum FP use (Table 4).

**Table 4:** Knowledge of study participants about postpartum family planning in Dilla Town, southern, Ethiopia, 2020

Knowledge factors		Frequency	percent
know contraceptive used to prevent unwanted pregnancy	Yes	564	97.9%
know contraceptive used to space pregnancy	Yes	532	92.4%
know contraceptive used to limit pregnancy	Yes	401	69.6%
Know time to initiate postpartum family planning	Yes	118	20.5%
know modern family planning methods	Yes	166	28.8%
Know side effects of modern family planning methods	yes	72	12.5%
Know exclusive breastfeeding used as family planning	Yes	133	23.1%
Know that fertility resumed after stopping family planning	Yes	251	43.6%
Knowledge of postpartum family planning	Poor Knowledge	334	58%
	Good knowledge	242	42%

### Time to initiate postpartum modern FP use

**Fig. 1:**
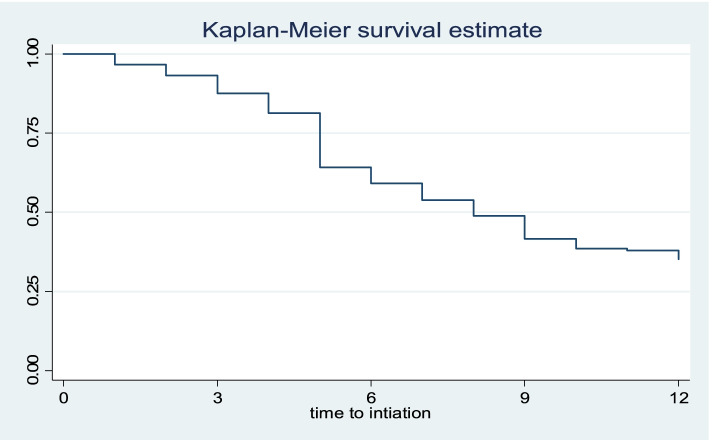
The Kaplan-Meier survival curve estimate time to initiate of postpartum modern contraceptive use among women of reproductive age group within the first 12 months of delivery in Dilla town, southern Ethiopia, 2020

In this study, 576 postpartum women were retrospectively followed for 12 months (1 year). The 200 cumulative proportion of postpartum modern contraceptive use was 383 (66.5%) with a 95% CI 201 (62.3–70.1%) at the end of 12 months. While the remaining 193 (33.5%) were rightly censored as the time of the follow up study period. Within the first 12 months of delivery, the median time for women of reproductive age to begin postpartum modern contraception was 7 months (IQR: 6, 8). Our findings revealed that only 11.2% of the postpartum women followed up started to use postpartum modern contraceptives by the second month after delivery. The proportion of users then increased steadily over the months, reaching 43.7, 60.4, and 65.5% at 6, 9, and 12 months, respectively (Fig. 1).

### Predictors of the time to initiate postpartum modern contraceptive use

After adjustment for possible confounders in multivariable Cox proportional hazard regression analysis, Education level of women, decide jointly on FP use, menses resumption, knowledge of postpartum family planning, number of ANC visits, distance to health facility and FP counseling during PNC service have significant association with the time to initiate postpartum modern contraceptive use. Women with a primary education were 3.01 [AHR = 3.01 (95% CI = 1.32–6.83)] times more likely than women with no formal education to begin postpartum modern contraception early. When compared to their counterpart, women who decide jointly with their partner are 8.85 [AHR = 8.85 (95% CI: =5.00–15.65] times more likely to initiate postpartum modern contraceptive use early. Postpartum women whose menses had returned were 9.24 [AHR = 9.24 (95% CI: 4.95, 17.25)] times more likely to initiate postpartum modern contraceptives early than their counterparts. Knowledge of PPFP Women who had good knowledge of postpartum family planning were positively associated with having the time to initiate postpartum modern contraceptive use after delivery. Women who had good knowledge of postpartum family planning were 1.56 [AHR = 1.56, (95% CI: 1.20, 2.02)] times more likely to initiate postpartum modern contraceptives early than women who had poor knowledge of PPFP. In terms of the time it takes to get to a medical facility, Women who traveled less than 0.5 hours to reach a health facility were 1.75 [AHR = 1.75, (95% CI: 1.05, 2.92)] times more likely than 228 women who traveled more than 1 hour to reach a health facility to begin postpartum modern 229 contraception early. Those women who had a number of ANC visits were 2.47 [AHR = 2.47, (95% CI: 1.17–5.19)] times more likely to initiate postpartum modern contraceptives early as compared to those women who had only a single number of ANC visits during their last pregnancy. FP counseling during PNC service was also found to be one of the predictors of time to initiate postpartum modern contraceptive use after delivery. The rate of using postpartum modern contraceptive use was 2.22 [AHR = 2.22 (95% CI: 1.46–3.38)] times shorter among women who received counseling service about FP during PNC service than among those women who weren’t counseled (Table 5).

**Table 5:** Bivariate and Multivariate Cox proportional Hazard regression analysis for factors associated with time to initiate of postpartum modern contraceptive use, in Dilla Town, southern Ethiopia, 2020

Variables	Use of postpartum modern contraceptive		Hazard ratio (95% CI)	
	Yes (user)	No (censored)	Unadjusted (CHR)	Adjusted (AHR)
**Educational level of women**				
No formal education	18 (3.1%)	17 (3.0%)	1	1
Read and write	16 (2.8%)	24 (4.2%)	1,09 (0.89, 1.64)**	3.01 (1.32, 6.83)***
Primary complete	168(29.2%)	121 (21.0%)	1.49 (0.92, 2.43)***	2.90 (1.53, 5.48)***
Secondary and above	181(31.4%)	31(5.4%)	2.98 (1.83, 4.86)***	2.66 (1.37, 5.18)***
**Occupation status of women**				
Housewife	149(25.9%)	131(22.7%)	1	1
Government employee	129(22.4%)	5(0.9%)	4.16 (3.26, 5.31)***	1.15 (0.80, 1.66)
Private employee	30 (5.2%)	47 (8.2%)	2.52 (1.68, 3.77)***	0.84 (0.51, 1.39)
Merchant	70 (12.2%)	5 (0.9%)	3.80 (2.82, 5.11)***	1.26 (0.82, 1.95)
Others^a^	5 (0.9%)	5 (0.9%)	0.87(0.35, 2.12)	0.91 (0.355, 2.3)
**Fertility desire**				
Went before 2 years	39 (6.8%)	52 (9.0%)	1	1
Went after 2 years	282 (49.0%)	126 (21.9%)	2.08 (1.49, 2.91)***	0.90 (0.53, 1.54)
Went no more children	61(10.6%)	15 (2.6%)	2.46 (1.64, 3.68)***	1.04 (0.71, 1.53)
**Discus with partner on modern family planning**				
No	179(31.1%)	172(29.9%)	1	1
Yes	204(35.4%)	21(3.6%)	3.76 (3.05, 4.64)**	0.84 (0.60, 1.19)
**Decide on use of modern family planning**				
Respondent (women)	53 (9.2%)	161 (28.0%)	1	1
Husband	115(20.0%)	22(3.8%)	4.89 (3.52, 6.79) **	9.19 (5.84, 14.4) ***
Both	215(37.3%)	10(1.7%)	10.1 (7.44, 13.9)**	8.85 (5.00,15.65) ***
**Menstruation resumed after recent birth**				
No	43(7.4%)	94(16.4%)	1	1
Yes	343(59.5%)	96(16.7%)	4.23 (3.07,5.82)***	9.24(4.95, 17.25)***
**Sexual intercourse resumed after recent birth**				
No	69 (12.0%)	109 (18.9%)	1	1
Yes	314(54.5%)	84(14.6%)	3.28 (2.52,4.26)***	0.86 (0.51, 1.44)
**Number of ANC visit**				
1	25(4.5%)	90(16.1%)	1	1
2–3	204(36.5%)	95(17.0%)	4.90 (3.23,7.44)***	2.20(1.20, 4.06)**
4+	140(25.0%)	5(0.9%)	13.0(8.47, 20.2)***	2.47(1.17,5.19)**
**FP counseling during ANC service**				
No	88(15.7%)	110(19.6%)	1	1
Yes	286(51.1%)	75(13.4%)	3.09 (2.44, 3.91)***	1.28 (0.86, 1.90)
**Distance to health facility**				
Less than 0.5 hours	54 (9.4%)	54(9.4%)	1.46 (1.02, 2.09)**	1.75(1.05, 2.92)**
0.5–1 hours	260(45.1%)	45(7.8%)	3.08 (2.35,4.02)***	1.95(1.38, 2.74)***
Greater than 1 hours	69(12.0%)	94(16.3%)	1	1
**FP counseling during PNC service for current child**				
No	25(6.8%)	88(24.2%)	1	1
Yes	226**(**62.1%)	25(6.9%)	4.16 (3.37, 5.13)***	2.22 (1.46, 3.38)***
**FP counseling during child immunization**				
No	132(25.4%)	180(34.8%)	1	1
Yes	201**(**34.9%)	5(0.9%)	6.01(4.83,7.48)***	1.14 (0.69, 1.90)
**knowledge of women on postpartum modern family planning**				
Poor Knowledge	178(30.9%)	156(27.1%)	1	1
Good knowledge	205(35.6%)	37(6.4%)	2.54 (2.07, 3.11)**	1.56 (1.20, 2.02) ***

^a^Daily laborer & student, *p* value = ** < 0.05 *** < 0.001

## Discussion

According to this study, the median time to begin postpartum modern contraception among women of reproductive age within the first 12 months of delivery was 7 months. Women’s education level, deciding jointly on postpartum modern contraceptive use, menstrual resumption, knowledge of postpartum family planning, number of ANC visits, distance to health facility, and FP counseling during PNC service were factors associated with the time to begin postpartum modern contraception.

The median time to initiate postpartum modern contraceptives is in line with a study conducted in Nairobi Urban Slums, Kenya [22]. It has been reported that the median time to postpartum modern contraceptive use after delivery was 6 months. This finding indicates that a number of women are at a high risk of unwanted or unplanned pregnancies because of the late initiation of postpartum modern contraceptive use, which is much later than WHO recommendation [3].

This study finding is lower than the studies conducted in Gozamen district, northwest Ethiopia (3.2 months of mean time to initiate postpartum modern contraceptive use) [23] and Kebribeyah 252 Town, eastern Ethiopia (2–3 months of mean time to initiate postpartum modern contraceptive use) [16]. This difference might be due to statistical analysis method variation, as these studies calculated the mean as a measure of the average for the time of postpartum modern contraceptive initiation after childbirth.

However, this study finding is higher than the study conducted in Uganda, [20], which documented 19 months of median time to postpartum modern contraceptive use. The reason might be due to target population variation, as the studies in Uganda included women who had given birth within the 5 years that increased the median time to postpartum modern contraceptive use after childbirth.

In this study, the cumulative proportion of postpartum modern contraceptive use among women of reproductive age was found to be 66.5% with a 95% CI (62.3–70.1%) at the end of 12 months of follow up. This finding is in line with a study conducted in Ganta-Afeshum district, Tigray, Ethiopia (68.1%) [24] and Debre Tabor town, North West Ethiopia (63%) [25]. This similarity might be due to the resemblance in socio-demographic characteristics and the time period of study in both reports, which assess the prevalence of postpartum modern contraceptive use after 12 months of study.

This finding is higher than that of studies conducted among postpartum women in Nigeria (8.32%), [26], Dabat district, northwest Ethiopia (10.3%), [27] and Gondar Town, Northwest Ethiopia (45.8%) [21]. The possible explanation for this variation may 270 be due to improvement in health service delivery, differences in study period, as well as the 271 socio-demographic status of the study participants.

However, this finding is lower than the study carried out in Addis Ababa, Ethiopia (80.3%) [15] Hossana town (73.9%), South Ethiopia (73.9%), and Kenya (86.3%). This difference might be due to differences in the study area’s socio demographic characteristics, cultural variations, and service accessibility. The study in Addis Ababa was done among urban residents, and such participants could have better access to information and health education.

This study shows that women who had primary education were 3 times more likely to initiate postpartum modern contraceptives early than those with no formal education. This finding is consistent with other studies carried out in rural Kenya [28] and Debre Tabor town, Northern Ethiopia [29]. This might be due to the fact that educated women have a better understanding of the benefits of modern contraceptives and the risks of short interval pregnancies. They also have a better inclination to visit health institutions and get the service than those with no formal education.

Postpartum women who decide jointly with their partner on postpartum modern contraceptive use are 8.8 times more likely to initiate postpartum modern contraceptive use earlier than those women who decide by themselves. This finding is also supported by other studies done in Gozamen District, Ethiopia [23], Gondar Town, northwest Ethiopia [21], and Arroresa district, Southern Ethiopia [18]. The reason for this finding could be the fact that decisions made jointly with agreement will have a better outcome regarding the use of postpartum contraceptives as compared with decisions made by only one side since the issue of family planning is not only the concern of one partner.

Women whose menses returned after delivery were 9 times more likely to initiate postpartum modern contraceptive use earlier than those women who hadn’t seen menses after their last delivery. This finding is consistent with studies done in Addis Ababa, Ethiopia [15], Gozamen district, Ethiopia [23], and Gondar Town, northwest Ethiopia [21]. The reason for this may be that menses resumption may make women aware of their fertility returning, which motivates them to start postpartum modern contraceptive use early. Moreover, different studies indicated that menses returning after birth was found to be a stimulating factor affecting the use of postpartum modern contraceptives and the absence of menses as the main factor for not using postpartum modern contraceptives.

Postpartum women who had good knowledge of postpartum family planning were 1.5 times more likely to initiate postpartum modern contraceptive use earlier than women who had poor knowledge of postpartum family planning. This finding is in line with previous reports from KebriBeyah Town, Eastern Ethiopia [16], Gondar Town, Northwest Ethiopia [21], and urban Ghana [19]. This is explained by the fact that knowledge determines the use of postpartum modern contraceptives.

The findings of this study show that mothers who had four or more ANC visits during their last pregnancy were 2.4 times more likely to use postpartum modern contraceptives early compared to those women who had only one ANC visit during their last pregnancy. This finding is consistent with studies from Kenya [30] and Uganda [20]. This could be due to the fact that mothers who attended more ANC visits had an opportunity to communicate with providers and to receive counseling regarding postpartum modern contraceptive use.

The present study revealed a significant difference in time to postpartum modern contraceptive use based on distance to a health facility. Women who had traveled less than 0.5 hours to reach a health facility were 1.7 times more likely to initiate postpartum modern contraceptive use early as compared to those women who had traveled more than 1 hour to reach a health facility. This finding is consistent with studies carried out in the rural Tigray region of northern Ethiopia [31]. The explanation for this finding is that the proximity of women to health facilities created an opportunity to use postpartum modern contraceptives.

Women who received FP counseling during PNC service were 2.2 times more likely to initiate postpartum modern contraceptive use early compared with their counterpart women. This finding is in line with a study conducted in Debre Tabor town, northern, Ethiopia [29]. This might be due to the fact that postnatal care visits give the opportunity to get more information and counseling from health professionals and can help postpartum women use modern contraceptive methods in an effective and timely manner.

The limitation of this study was that due to the retrospective nature of the study, there might be a recall bias might have been introduced on some of the questions that required the women to recall past information. Another limitation is that women who have lost appointment cards or family planning to check the date of start. Reporting inaccurate information to an interviewer in order to please him or her could also result in a social desirability bias. It mainly focuses on individual level factors, socio-cultural factors, and the male partner’s involvement related factors not assessed in this study.

## Conclusions

This study found that postpartum modern contraceptive use among women of reproductive age was delayed longer than compared to the WHO recommendation within the first 12 months of delivery. Women’s education level, deciding on family planning use jointly with their partner, menstrual resumption, knowledge of postpartum family planning, number of ANC visits during last pregnancy, distance to health facility, and family planning counseling during PNC services were factors that positively correlated with the time to begin postpartum modern contraception.

Based on our finding we recommend that, the Dilla town health office, should provide support and encourage basic education for all women who have not attended formal education to improve the late initiation of the FP services in collaboration with the education bureau.

Health facilities and health professionals should be support pregnant women to increase the number of antenatal care visits. Further we suggest that for those researchers who are interested should conduct prospective cohort design by including other factors such as gender norms, socio cultural factors, and male partner-related factors that hinder postpartum modern contraceptive use.

## Data Availability

The data sets used during the current study are available from the corresponding author on reasonable request.

## Abbreviations

ANC: Antenatal Care
AHR: Adjusted Hazard Ratio
CHR: Crude Hazard Ratio
CSA: Central Statistical Agency
DHS: Demographic Health Survey
EDHS: Ethiopian Demographic and Health Survey
FP: Family Planning
HIV: Human Immune Deficiency virus
IUCD: Intra Uterine Contraceptive Device
KM: Kilo meter
LAM: Lactational Amenorrhea
PCA: Principal Component Analysis
PNC: Post Natal Care
POP: Progesterone Only Pill
PPFP: Postpartum Family Planning
RR: Relative Risk
SSA: Sub-Saharan Africa
STI: Sexual Transmitted Infection
STD: Sexually Transmitted Diseases
WHO: World Health Organization

## References

[1] World Health Organization (2013). Programming strategies for postpartum family planning.

[2] World Health Organization (2005). Report of a WHO technical consultation on birth spacing. WHO/RH.

[3] World Health Organization (2013). WHO recommendations on postnatal care of the mother and newborn: World Health Organization. WHO Maternal Child.

[4] Ross JA, Winfrey WL (2012). Unmet need for contraception in the developing world and the former Soviet Union: an updated estimate. Int Fam Plan Perspect.

[5] Ahmed S, Li Q, Liu L, Tsui AO (2012). Maternal deaths averted by contraceptive use: An analysis of 172 countries. Lancet.

[6] Africa WHO, UNICEF, UNFPA, World Bank Group and the United Nations Population Division (2016). Trends in Maternal Mortality Estimates from 1990 to 2015. WHO/RH.

[7] Sedgh G, Singh S, Husain R (2014). Intended and unintended pregnancies worldwide in 2012 and recent trends. Stud Fam Plan.

[8] United Nations (UN) (2009). “World contraceptive use 2011”. New York: UN Department of Economic and Social Affairs. Vernon, Ricardo. “Meeting the family planning needs of postpartum women”. Stud Fam Plan.

[9] Rossier C, Bradley SE, Ross J, Winfrey W (2015). Reassessing unmet need for family planning in the postpartum period. Stud Fam Plan.

[10] Moore Z (2015). Missed opportunities for family planning: an analysis of pregnancy risk and contraceptive method use among postpartum women in 21 low- and middle-income countries. Contraception.

[11] Central Statistical Agency (CSA) and ICF International. Ethiopia Demographic and Health Survey. Ethiopian Demographic and Health Survey. 2016;1(4):1–36.

[12] Mumah J, Kazuyo M, Mutua M (2015). Contraceptive adoption, discontinuation, and switching among postpartum women in Nairobi’s urban slums. Stud Fam Plan.

[13] Fagbamigbe AF, Adebowale AS (2015). Survival analysis of time to uptake of modern contraceptives among sexually active women of reproductive age in Nigeria. BMJ Open.

[14] Gebreselassie T, Rutstein SO, Mishra V (2008). Contraceptive use, breastfeeding, amenorrhea and abstinence during the postpartum period: an analysis of four countries. DHS Analytical Studies.

[15] Gebremedhin AY, Kebede Y, Gelagay AA, Habitu YA (2018). Family planning use and its associated factors among women in the extended postpartum period in Addis Ababa, Ethiopia. Contracept Reprod Med.

[16] Nigussie AT, Girma D, Tura G (2016). Postpartum family planning utilization and associated factors among women who gave birth in the past 12 months, Kebri Beyah town, Somali region, eastern Ethiopia. J Women's Health Care.

[17] Abdulbasit M, Nega F, Fitsum W, Habtamu M, Zelalem T (2016). Factor associated with experience of modern contraceptive use before pregnancy among women who gave birth in Kersa HDSS, Ethiopia. BMC Public Health.

[18] Aregahegn D, Muluemebet A, Tsedach A, Dawit H (2018). Timely initiation of postpartum contraceptive utilization and associated factors among women of child bearing age in Aroressa District, Southern Ethiopia. BMC Public Health.

[19] Coomson JI, Manu A (2019). Determinants of modern contraceptive use among postpartum women in two health facilities in urban Ghana: a cross-sectional study. Contracept Reprod Med.

[20] Wamala R, Kabagenyi A, Kasasa S. Predictors of time-to-contraceptive use from resumption of sexual intercourse after birth among women in Uganda. Int J Popul Res. 2017;1-12.

[21] Berta M, Feleke A, Abate T, Worku T, Gebrecherkos T (2018). Utilization and associated factors of modern contraceptives during extended postpartum period among women who gave birth in the last 12 months in Gondar Town, northwest Ethiopia. Ethiop J Health Sci.

[22] Ndugwa RP (2011). Menstrual pattern, sexual behaviors, and contraceptive use among postpartum women in Nairobi urban slums. J Urban Health.

[23] Gizaw W, Zewdu F, Abuhay M, Bayu H (2017). Extended Postpartum Modern Contraceptive Utilization and Associated Factors among Women in Gozamen District, East Gojam Zone, Northwest Ethiopia, 2014. Insights Reprod Med.

[24] Gebremariam A, Gebremariam H (2017). Contraceptive use among lactating women in Ganta Afeshum District, Eastern Tigray, Northern Ethiopia, 2015: a cross sectional study. BMC Pregnancy Childbirth.

[25] Taye EB, Mekonen DG, Tibeb Zena D (2018). Prevalence of post partum modern family planning utilization and associated factors among postpartum 4mothers in Debre Tabor town, North West Ethiopia. BMC Res Notes.

[26] Akinlo A, Bisiriyu A, Esimai O (2013). Influence of Use of Maternal Health Care on Postpartum Contraception in Nigeria.

[27] Zelalem B, Abebaw G, Senafikish A (2015). Contraceptive adoption in the extended postpartum period is low in Northwest Ethiopia. BMC Pregnancy Childbirth.

[28] Rose J, Faith T, Barasa SO, Peter N (2017). Determinants of contraceptive use among postpartum women in a county hospital in rural Kenya. BMC Public Health.

[29] Abraha TH, Teferra AS, Gelagay AA (2017). Postpartum modern contraceptive use innorthern Ethiopia: prevalence and associated factors. Epidemiol Health.

[30] Do M, Hotchkiss D (2013). Relationships between antenatal and postnatal care and post-partum modern contraceptive use: evidence from population surveys in Kenya and Zambia. BMC Health Serv Res.

[31] Abraha (2018). Predictors of postpartum contraceptive use in rural Tigray region, northern Ethiopia: a multilevel analysis. BMC Public Health.

